# Ascertaining invasive breast cancer cases; the validity of administrative and self-reported data sources in Australia

**DOI:** 10.1186/1471-2288-13-17

**Published:** 2013-02-11

**Authors:** Anna Kemp, David B Preen, Christobel Saunders, C D’Arcy J Holman, Max Bulsara, Kris Rogers, Elizabeth E Roughead

**Affiliations:** 1 Centre for Health Services Research, School of Population Health, The University of Western Australia, 35 Stirling Hwy, Crawley, WA, 6009, Australia; 2 Illawarra Health and Medical Research Institute, Building 32, University of Wollongong, Wollongong, NSW, 2522, Australia; 3 School of Surgery, The University of Western Australia, 35 Stirling Hwy, Crawley, WA, 6009, Australia; 4 School of Population Health, The University of Western Australia, 35 Stirling Hwy, Crawley, WA, 6009, Australia; 5 Institute of Health and Rehabilitation Research, University of Notre Dame, PO Box 1225, Fremantle, WA, 6959, Australia; 6 Prevention Research Collaboration, Sydney School of Public Health, University Fisher Road, Sydney, NSW, 2206, Australia; 7 Quality Use of Medicines and Pharmacy Research Centre, School of Pharmacy and Medical Sciences, University of South Australia, GPO Box 2471, Adelaide, SA, 5001, Australia

**Keywords:** 45 and up study, Sensitivity, Specificity, Positive predictive value, Lumpectomy, Mastectomy, Radiotherapy, Hospital diagnosis, Tamoxifen, Anastrazole, Self-report

## Abstract

**Background:**

Statutory State-based cancer registries are considered the ‘gold standard’ for researchers identifying cancer cases in Australia, but research using self-report or administrative health datasets (e.g. hospital records) may not have linkage to a Cancer Registry and need to identify cases. This study investigated the validity of administrative and self-reported data compared with records in a State-wide Cancer Registry in identifying invasive breast cancer cases.

**Methods:**

Cases of invasive breast cancer recorded on the New South Wales (NSW) Cancer Registry between July 2004 and December 2008 (the study period) were identified for women in the 45 and Up Study. Registry cases were separately compared with suspected cases ascertained from: i) administrative hospital separations records; ii) outpatient medical service claims; iii) prescription medicines claims; and iv) the 45 and Up Study baseline survey. Ascertainment flags included diagnosis codes, surgeries (e.g. lumpectomy), services (e.g. radiotherapy), and medicines used for breast cancer, as well as self-reported diagnosis. Positive predictive value (PPV), sensitivity and specificity were calculated for flags within individual datasets, and for combinations of flags across multiple datasets.

**Results:**

Of 143,010 women in the 45 and Up Study, 2039 (1.4%) had an invasive breast tumour recorded on the NSW Cancer Registry during the study period. All of the breast cancer flags examined had high specificity (>97.5%). Of the flags from individual datasets, hospital-derived ‘lumpectomy and diagnosis of invasive breast cancer’ and ‘(lumpectomy or mastectomy) and diagnosis of invasive breast cancer’ had the greatest PPV (89% and 88%, respectively); the later having greater sensitivity (59% and 82%, respectively). The flag with the highest sensitivity and PPV ≥ 85% was 'diagnosis of invasive breast cancer' (both 86%). Self-reported breast cancer diagnosis had a PPV of 50% and sensitivity of 85%, and breast radiotherapy had a PPV of 73% and a sensitivity of 58% compared with Cancer Registry records. The combination of flags with the greatest PPV and sensitivity was ‘(lumpectomy or mastectomy) and (diagnosis of invasive breast cancer or breast radiotherapy)’ (PPV and sensitivity 83%).

**Conclusions:**

In the absence of Cancer Registry data, administrative and self-reported data can be used to accurately identify cases of invasive breast cancer for sample identification, removing cases from a sample, or risk adjustment. Invasive breast cancer can be accurately identified using hospital-derived diagnosis alone or in combination with surgeries and breast radiotherapy.

## Background

Routinely-collected and self-reported health data are increasingly used to identify health status and service use in research. In Australia, State-based statutory cancer registries are considered the ‘gold standard’ for identifying breast cancer cases for research purposes and in recent years these data have been linked to other routinely-collected datasets for research [1-3].

Since December 2008, delays in release of mortality data from the Australian Bureau of Statistics have prevented the New South Wales (NSW) Cancer Registry from releasing data [4]. Consequently, the gold-standard dataset for identifying breast cancer in NSW has been inaccessible from 2009 onward and cancer researchers cannot ascertain cases from this source. Aside from these recent Australian issues, researchers in many countries face lengthy delays, cost or political barriers to accessing linked, routinely-collected datasets, which are often held by separate custodians and cover different jurisdictions [5-8]. Researchers who only have access to single datasets (e.g. hospital records), or specified packages of automatically linked datasets (e.g. English national hospital and death records [9], Australian medical service and prescription claims linked with NSW 45 and Up Study [10]) may want to identify cases of breast cancer without linkage to a Cancer Registry.

The aim of this study was to determine whether incident cases of invasive breast cancer can be accurately ascertained through a range of routinely-collected administrative and self-reported health datasets, with comparisons made to histologically-confirmed Cancer Registry records.

## Methods

### Study sample

The study sample was selected from participants enrolled in the 45 and Up Study; a cohort of approximately 267,000 adults aged ≥45 years residing in NSW [10]. Participants in this study provided demographic, lifestyle and health information upon joining the study and consented to having their routinely-collected health data linked and analysed for research purposes [11]. Baseline information for the 45 and Up Study cohort are already linked to medical service claims and pharmaceuticals publically-subsided by the Australian government. These datasets are now being used for many epidemiological studies e.g. [12-14]. Researchers can also apply to have these records linked to other NSW and national datasets on a project-by-project basis. Detailed information regarding the establishment and recruitment for the 45 and Up Study are described elsewhere [10]. The present study included 143,010 women recruited between January 2006 and April 2009, who had completed breast cancer-related items in the baseline survey of the 45 and Up Study.

### Ascertaining cases using the gold standard

The NSW Cancer Registry contains, by statutory requirement, records of all cancers diagnosed or treated in NSW [15,16]. The Cancer Registry was considered the ‘gold standard’ source for cancer identification in this study. Cases were defined as women with a diagnosis of invasive breast cancer listed on the NSW Cancer Registry during the study period; 1 July 2004 to 31^st^ December 2008. Codes used to identify cases were International Classification of Diseases version 10 with Australian modifications (ICD-10-AM) C50.0-C50.9 [17]. Participants with no registry record during the study period were considered non-cases.

### Data sources and linkage

We accessed unit-record linked data from: i) the 45 and Up Study baseline survey, ii) NSW Admitted Patient Data Collection; iii) Medicare Benefits Schedule (MBS) claims; and iv) Pharmaceutical Benefits Scheme (PBS) claims. All data linkage was conducted by the NSW Centre for Health Record Linkage [18] and researchers were provided with de-identified data only.

### Ascertaining cases using other datasets

Hospital diagnosis ascertainment flags were identified through the NSW Admitted Patient Data Collection. This dataset captures all admissions to public and private hospitals in the State of NSW. As with the Cancer Registry, we identified participants with a principal inpatient diagnosis of invasive breast cancer using ICD-10-AM codes C50.0-C50.9. Suspected cases flagged by inpatient diagnosis were defined as true positives if they occurred within three months of the Cancer Registry date of diagnosis. Flags for breast cancer surgeries were also identified from hospital data. We used ICD-10-AM procedure codes to identify mastectomy (31518–00, 31518–01, 31524–00, 31524–01), and excision of malignant breast lump (lumpectomy) (31500–00, 31500-01, 31503-00, 31503-01, 31506-00, 31506-01, 31509-00, 31509-01, 31512-00, 31512-01) [17]. Suspected cases flagged by surgeries occurring within three months of the Cancer Registry date of diagnosis were considered to be true positives.

Flags for breast radiotherapy and prescription medicines were identified through the MBS and PBS datasets. The MBS is a claims database which captures medical services subsidised by the Australian Federal Government for all Australian citizens [19]. As with the MBS, the PBS is a national scheme covering all Australian citizens [20]. Breast radiotherapy is conducted on an outpatient basis in NSW and was not detected in the hospital dataset. We identified claims for breast radiotherapy using MBS codes 15221, 15236, 15251, and 15266 [21]. We identified claims for dispensings of prescribed medicines used to treat breast cancer using PBS codes. These datasets captured the date of service for radiotherapy and dispensing of medicines. These medicines included selective oestrogen reuptake inhibitors (tamoxifen 2109B, 2110C and toremifene 8216K), aromatase inhibitors (anastrozole 8179L, exemestane 8506Q, letrozole 8245Y); and other breast cancer therapies (goserelin 1452M; trastuzumab 4632T, 4639E, 4650R, 4703M, 7264H, 7265J, 7266K, 7267L; lapatinib 9148L; 500 mg preparations of medroxyprogesterone 2728N) [22]. All these therapies are only subsidized for use in women breast cancer. Only 500 mg preparations of medroxyprogesterone were included because lower dose preparations are subsidised for indications other than breast cancer in Australia [23]. Suspected cases of invasive breast cancer flagged by breast radiotherapy or the specified medicines were considered to be true positives if these services were provided within 12 months of the Cancer Registry date of diagnosis. The follow up periods for diagnosis, surgery, radiotherapy and prescription medicines vary and were selected to allow for the usual delays in treatment after diagnosis and were determined from sensitivity analysis examining different follow up periods (Additional file 1: Sensitivity analyses).

### Process for comparing self-reported diagnosis with the cancer registry

Self-reported diagnosis of breast cancer was identified from the 45 and Up Study baseline survey. Recruitment to the study and completion of the baseline survey commenced in January 2006, making this the latest date where all participants would uniformly have the opportunity to self-report a diagnosis of breast cancer. Therefore, self-reports were only compared against cases in the Cancer Registry for the period 1 July 2004 to 31^st^ December 2005.

**Figure 1:**
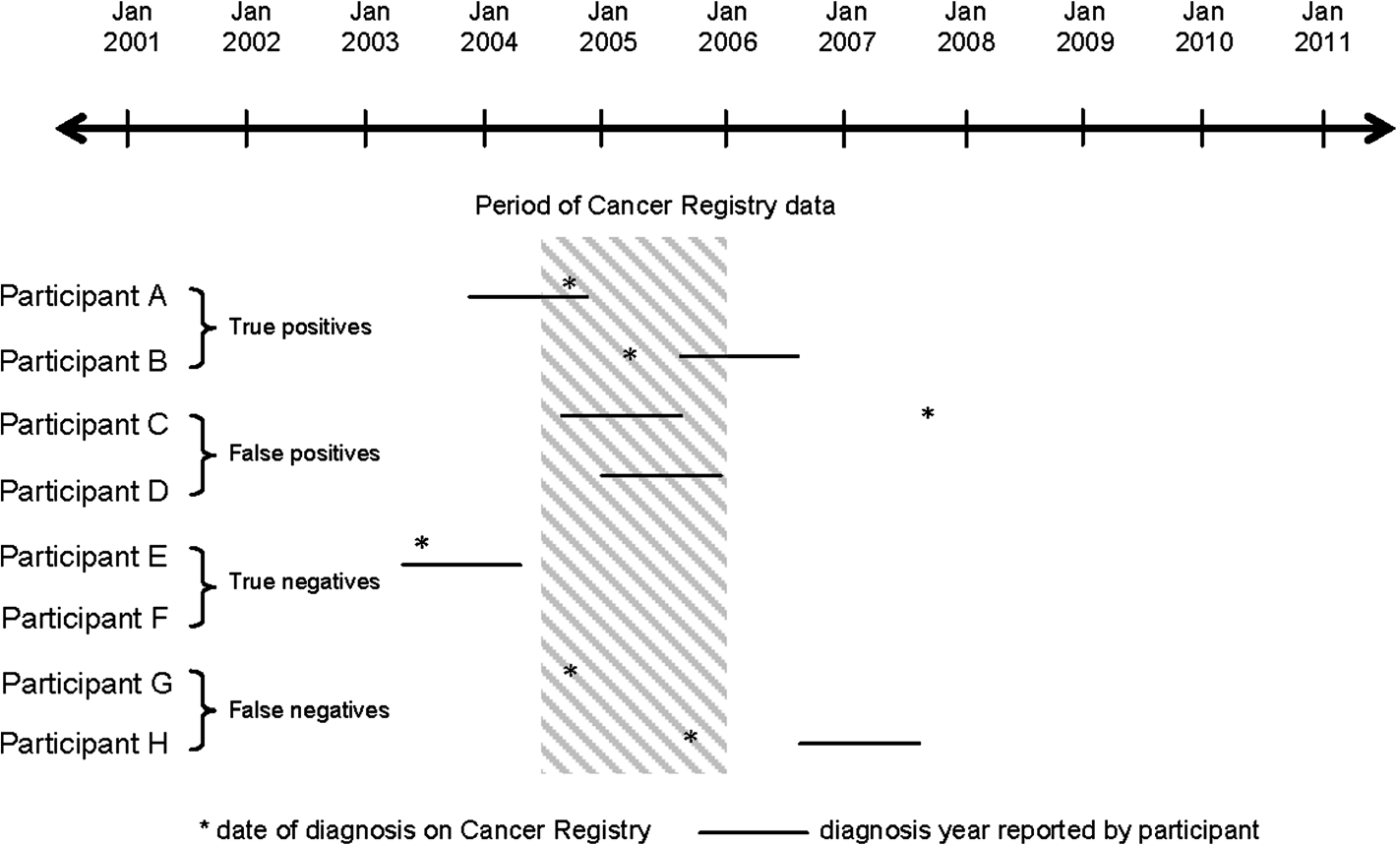
Possible relationships between the study period, diagnosis date recorded on the Cancer Registry, and the diagnosis year reported by participants.

The baseline survey asked participants to indicate their current age in years and months; whether a doctor had ever told them they had breast cancer (yes/no) and, if yes, their age in years at diagnosis. We then calculated the 12 month period in which the participant was the age they reported being at diagnosis. For example, a woman aged 72 years and 4 months when recruited to the study on 19^th^ August 2008 and reporting a cancer diagnosis at age 68 would have a proxy ‘diagnosis year’ from April 2004 to March 2005. A true positive was defined as a self-reported diagnosis year overlapping the period July 2004-December 2005, and a Cancer Registry date of diagnosis during this period (see Figure 1, Participants A and B). A false positive was defined as occurring when the reported diagnosis year overlapped the period July 2004-December 2005 but no Cancer Registry record was found for the period (Participants C and D). A true negative was defined as occurring when the participant did not report a breast cancer diagnosis or reported a diagnosis year that did not overlap the period, and no Cancer Registry record was found for the period (Participants E and F). A false negative was defined as occurring when a participant did not report a diagnosis of breast cancer or reported a diagnosis year which did not overlap with the period and a Cancer Registry record was found for the period (Participants G and H).

#### Statistical analyses

Breast cancer flags drawn from individual datasets were compared against the Cancer Registry for PPV, sensitivity, and specificity (see Table 1) [24,25]. Researchers will prioritise these indicators differently depending on their reason for identifying cases (e.g. to define a sample, exclude cases from a sample, or for risk adjustment). We sought to identify flags with high PPV (≥85%) and within that, the greatest sensitivity. We intend to use these flags to identify a sample of women with invasive breast cancer for future studies.

**Table 1:** Validity of breast cancer flags in individual datasets compared with the Cancer Registry, July 2004-December 2008

**Data set**	**Breast cancer flags**	**Positive predictive value**	**Sensitivity**	**Specificity**
45 and Up baseline survey^1^	Self-reported diagnosis of breast cancer ^2^ within a year of birth-year reported	49.8%	84.7%	99.6%
Admitted Patient Data Collection	Diagnosis^3^ of invasive breast cancer	85.9%	86.1%	99.8%
	Lumpectomy	52.0%	60.7%	99.2%
	Mastectomy	70.8%	32.6%	99.8%
	Lumpectomy OR mastectomy	56.4%	84.4%	99.1%
	Lumpectomy AND diagnosis of invasive breast cancer	89.0%	59.1%	99.9%
	Mastectomy AND diagnosis of invasive breast cancer	85.4%	31.8%	99.1%
	(Lumpectomy or mastectomy) AND diagnosis of invasive breast cancer	87.7%	82.3%	99.8%
	(Lumpectomy or mastectomy) OR diagnosis of invasive breast cancer	56.5%	88.2%	99.0%
	Mastectomy OR diagnosis of invasive breast cancer	79.7%	87.6%	99.7%
	Lumpectomy OR diagnosis of invasive breast cancer	58.2%	87.6%	99.1%
Medicare Benefits Schedule	Breast radiotherapy	72.8%	57.6%	99.7%
Pharmaceutical Benefits Scheme	Dispensed medicine for breast cancer ^4^	45.5%	65.4%	98.9%

1: Comparison to Cancer Registry was restricted to July 2004-December 2005.

2: Reported cancer with birth-year occurring in or overlapping with the period July 2004-December 2005.

3: Primary diagnosis field.

4: Tamoxifen, toremifene, anastrozole, exemestane, letrozole, goserelin, trastuzumab, lapatinib, and medroxyprogesterone 500 mg.

To determine if combinations of flags improved PPV, sensitivity or specificity, clinically meaningful combinations of flags were determined in consultation with a breast cancer surgeon and medical oncologist. These combinations of flags (as described in Table 2) were derived from the commonly utilised combination of 45 and Up Study baseline, MBS and PBS data; and from all of the available datasets (hospital, 45 and Up Study baseline data, MBS and PBS data). These combination flags were also assessed against the Cancer Registry for PPV, sensitivity and specificity. Additional sensitivity analyses were undertaken to determine how many ‘false reports’ of breast cancer on the 45 and Up Study baseline survey were recorded cases on the Cancer Registry, but with incorrectly reported age at diagnosis. All analyses were conducted in IBM SPSS version 19.0.

**Table 2:** Validity of combinations of breast cancer flags compared with the Cancer Registry, July 2004-December 2008

**Breast cancer flags**	**Positive predictive value**	**Sensitivity**	**Specificity**
**45 and Up Study baseline, Medicare Benefits Schedule and Pharmaceutical Benefits Scheme**			
Breast radiotherapy AND dispensed medicine	80.1%	40.3%	99.9%
Breast radiotherapy OR dispensed medicine^1^	47.9%	82.7%	98.7%
Breast radiotherapy AND self-reported diagnosis	69.3%	36.1%	99.9%
Breast radiotherapy AND dispensed medicine AND self-reported diagnosis^2^	72.1%	23.9%	99.9%
(Breast radiotherapy OR dispensed medicine) AND self-reported diagnosis	58.6%	60.4%	99.8%
Breast radiotherapy OR dispensed medicine OR self-reported diagnosis	27.0%	95.3%	97.7%
**Admitted Patients Data Collection, 45 and Up Study baseline**, **Medicare Benefits Schedule and Pharmaceutical Benefits Scheme**			
(Lumpectomy or mastectomy) AND diagnosis of invasive breast cancer AND breast radiotherapy	90.1%	47.5%	99.9%
(Lumpectomy or mastectomy) AND diagnosis of invasive breast cancer AND breast radiotherapy AND dispensed medicine	89.7%	33.7%	99.9%
(Lumpectomy or mastectomy) AND diagnosis of invasive breast cancer AND dispensed medicine	87.8%	55.0%	99.9%
(Lumpectomy or mastectomy) AND (diagnosis of invasive breast cancer OR breast radiotherapy)	83.0%	83.2%	99.8%
(Lumpectomy or mastectomy) AND (diagnosis of invasive breast cancer OR dispensed medicine)	84.1%	83.1%	99.8%
(Lumpectomy or mastectomy) AND (diagnosis of invasive breast cancer OR breast radiotherapy OR dispensed medicine)	80.5%	83.6%	99.7%
(Lumpectomy or mastectomy) AND diagnosis of invasive breast cancer AND self-reported diagnosis	88.5%	71.1%	99.9%
(Lumpectomy or mastectomy) AND diagnosis of invasive breast cancer AND breast radiotherapy AND self-reported diagnosis	87.3%	29.4%	99.9%

1: Tamoxifen, toremifene, anastrozole, exemestane, letrozole, goserelin, trastuzumab, lapatinib, and medroxyprogesterone 500 mg.

2: Flag combinations including self-reported diagnosis of breast cancer were compared against the Cancer Registry for the period July 2004 to December 2005.

#### Ethics approval

Ethical approval for this project was received from The University of Western Australia (WA) Human Research Ethics Committee (approval RA/4/1/4589) and the NSW Population and Health Services Research Ethics Committee (approval HREC/11/CIPHS/35).

## Results

Of the 143,010 women in the 45 and Up Study cohort, 2039 (1.4%) had an invasive breast tumour recorded on the NSW Cancer Registry during the study period. Of these, 681 (33.4%) occurred between 1 July 2004 and 31^st^ December 2005, and this subgroup was compared against self-reported breast cancer for the cohort.

### Breast cancer flags from individual datasets

Table 1 shows the number of suspected cases flagged within each of the datasets examined. All of the breast cancer flags had high specificity (>98.5%). Self-reported diagnosis of breast cancer had a PPV of only 50% compared with the Cancer Registry; however the sensitivity was 85%. PPV and sensitivity of the hospital diagnosis of invasive breast cancer were both 86% and for lumpectomy 52% and 61%, respectively. Hospital-derived mastectomy had a higher PPV of 71% against the Cancer Registry but lower sensitivity (33%). When considering combinations of flags within hospital data, the one with the highest sensitivity for a flag with PPV over 85% was ‘(lumpectomy or mastectomy) and diagnosis of invasive breast cancer’. PPV and sensitivity for this combination were 88% and 82%, respectively. Among the flags from medical service and prescription medicine claims, breast radiotherapy had the highest PPV and sensitivity (73% and 58%, respectively). Use of any medicine for breast cancer had a PPV of 46% and sensitivity of 65%.

### Breast cancer flags from multiple datasets

Combinations of flags derived from the package of 45 and Up Study baseline survey, MBS and PBS datasets are shown in Table 2. All of the ascertainment flags from multiple datasets had high specificity (>97.5%). None of the combinations of flags from these datasets had PPV >85%; the highest PPV being 80% for the flag combination ‘breast radiotherapy and a dispensed medicine (sensitivity 40%). Very high sensitivity was observed for the flag combination of ‘breast radiotherapy or a dispensed medicine or self-reported diagnosis of breast cancer' (95%); however PPV was low (27%).

Combinations of flags which included hospital data are also shown in Table 2. Specificity was above 99.5% for all the flag combinations. Good PPV (>85%) was found for several flag combinations including surgeries, hospital diagnosis, breast radiotherapy, dispensed medicines, or self-reported diagnosis; however sensitivity was lower (range 29%-71%). The combination of flags with the highest sensitivity and PPV over 85% was ‘(lumpectomy or mastectomy) and hospital diagnosis and self-reported diagnosis)’ (PPV 89%, sensitivity 41%). In contrast to the flags derived from hospital data alone none of the combinations of flags from multiple datasets were found to have PPV ≥85% and sensitivity above 80%.

A total of 581 women were considered to have ‘falsely’ reported a diagnosis of breast cancer because they had no record of invasive breast cancer on the Cancer Registry within 12 months of the birth year they reported. Of these, 399 (69%) were found to have a Cancer Registry record for invasive breast cancer for an earlier or later period. These women had misreported their age at diagnosis but not their history of diagnosis.

## Discussion

We sought to identify flags for invasive breast cancer with PPV ≥85% and, within that, the greatest sensitivity. Of the ascertainment flags examined from individual datasets, the flag meeting these criteria was hospital-derived ‘diagnosis of invasive breast cancer’. When compared with the gold-standard Cancer Registry this flag combination had a PPV and sensitivity both of 86%. In other words, 86% of the suspected cases identified by this flag were true positives, and 86% of the cases listed on the Cancer Registry during the study period were identified by this flag. The addition of flags from other Australian datasets (i.e. medical service, prescription claims and survey data) to these hospital-derived flags did not results in combinations with both PPV and sensitivity over 85%.

Researchers working with the combination of Australian medical service claims, pharmaceutical claims and self-reported data could most accurately identify cases of invasive breast cancer using the flag combination of ‘breast radiotherapy and a dispensed medicine’. Around 80% of cases identified by this flag were true cases, compared with the gold standard, and this flag identified 40% of the invasive breast cancers recorded on the Cancer Registry during the study period. Much higher sensitivity was achieved with the flag ‘breast radiotherapy or a dispensed medicine or self-reported diagnoses; however the corresponding PPV was poor (27%).

To our knowledge, this is the first study to examine the validity of multiple breast cancer flags from multiple datasets against an Australian State Cancer Registry. Such investigation is important due to the increasing use of administrative and self-reported data in epidemiological studies, and with the unavailability of Cancer Registry data in some jurisdictions. We have used health and medical records for a large, heterogeneous sample of women for whom all public and private inpatient diagnoses and surgeries, subsidised outpatient procedures and medicines have been captured.

Some limitations exist which may have implications for this study. This study was conducted as part of a larger program of research examining use of endocrine therapies for invasive breast cancer in Australian clinical practice. The data we requested from the Cancer Registry were therefore restricted to invasive breast cancer and did not include records for ductal carcinoma in situ (DCIS). We were therefore unable to determine how often false positive flags were picking up genuine cases of DCIS and how many were unrelated to breast cancer of any kind. We examined the validity of various breast cancer flags for women in the 45 and Up Study who, by definition, are aged 45 years and over and have consented to their health records being used for research purposes. The health service use of these women may differ from younger women with breast cancer, or women who do not agree to participate in cohort studies. Therefore, the PPV, sensitivity and specificity calculated here for various flags may differ from those that would be found in whole-of-population studies. The validity of the flags examined here are impacted by the proportion of women who move out of NSW between diagnosis and treatment, as well as those dying prior to treatment or declining treatment. It may also be that the validity of the breast cancer flags examined here will change over time in response to changes in health service use and medical advancement.

Each of the flags we examined had very high specificity, which is to be expected given the low prevalence of breast cancer within the cohort (1.4%). In such a scenario, even a model which predicted no breast cancer at all would retain high specificity. Therefore, it is important to examine the PPV and sensitivity of all predictors. The optimum method for identifying cases of breast cancer without access to a Cancer Registry will depend on the type and number of datasets available and the reason cases need to be identified. Researchers seeking to exclude possible cases of breast cancer from their datasets will be most concerned with the specificity of breast cancer flags. All of the breast cancer flags we examined in this study, whether derived from individual or multiple datasets, had high specificity (>97.5%). Each of these would be suitable for identifying non-cases with high accuracy. Researchers wishing to identify any suspected cases of breast cancer for situations where some false positives are acceptable, such as risk adjustment, would likely prioritise flags with high sensitivity. In contrast, PPV would likely be most important for researchers seeking to identify breast cancer cases with the fewest possible false negatives (e.g. to select an affected cohort) [26].

The sensitivity and specificity of the hospital-derived flags we calculated are similar to those reported in a NSW study, which demonstrated the hospital procedures ‘lumpectomy or mastectomy’ identified invasive breast cancers in the Cancer Registry with high sensitivity (83%) and specificity (95%) [27]. International studies have also reported high accuracy for hospital records in identifying breast cancer [26,28,29]. In an Italian study of hospital records, the combination of hospital diagnosis together with ‘lumpectomy or mastectomy’ accurately identified the majority of cases on the Cancer Registry (PPV 91%, sensitivity 85%, specificity 99%) [26].

We found that self-reported diagnosis of breast cancer correctly identified 50% of invasive breast cancer diagnoses to within 12-months of the birth year reported. While one would expect individuals to self-report diagnoses such as cancer reliably [30,31], the baseline survey did not ask woman to differentiate between invasive breast cancer and DCIS. Women may have accurately reported a DCIS as a diagnosis of breast cancer, however our data extract from the Cancer Registry was limited to invasive tumours so this was not able to be confirmed. In addition, women may not accurately recall the age at which they were diagnosed [30-32]. In this study, women reporting a ‘diagnosis year’ overlapping the period July 2004 to December 2005 but without a Cancer Registry diagnosis during this period were considered false positives. A sensitivity analysis indicated that 399 of 581 (69%) of these ‘false positives’ (according to our definition) did have a Cancer Registry diagnosis for invasive breast cancer, but had incorrectly reported their age at diagnosis.

## Conclusion

The Cancer Registry is the gold standard for identifying incident cases of invasive breast cancer in most jurisdictions. The findings from this study indicate that other administrative and self-reported datasets examined can be used to accurately identify cases of invasive breast cancer when Cancer Registry data are unavailable. Cases of invasive breast cancer were most accurately identified by hospital-derived diagnosis of invasive breast cancer. This flag would be most suitable for researchers seeking to identify a study cohort with invasive breast cancer or for risk adjustment [26]. However, all of the flags examined in this study accurately identified cases without invasive breast cancer, so are suitable for researchers wishing to exclude cases from population-based datasets likely to have low prevalence of breast cancer.

## Competing interests

The authors declare that they have no competing interests.

## Authors’ contributions

AK conceived of the study, performed the statistical analyses, interpreted data and drafted the manuscript. ER participated in the design of the study and interpretation of data and helped to write the manuscript. KR and MB aided with data interpretation and critically reviewed the manuscript. DP, CS and CDH assisted with acquisition of data and critically reviewed the manuscript. All authors read and approved the final manuscript.

## Pre-publication history

The pre-publication history for this paper can be accessed here:

http://www.biomedcentral.com/1471-2288/13/17/prepub

## Supplementary Material

Additional file 1 Sensitivity analyses comparing follow-up periods for selected flags, compared with the Cancer Registry, July 2004-December 2008. Click here for file
