# Supplementary material for: Ascertaining invasive breast cancer cases; the validity of administrative and self-reported data sources in Australia
Source: BMC Med Res Methodol. 2013 Feb 11;13:17. doi: 10.1186/1471-2288-13-17 (PMC3599953; doi:10.1186/1471-2288-13-17)
Supplement: Additional file 1 — Sensitivity analyses comparing follow-up periods for selected flags, compared with the Cancer Registry, July 2004-December 2008. [file 1471-2288-13-17-S1.docx]

| Appendix 1: Sensitivity analyses comparing follow-up periods for selected flags, compared with the Cancer Registry, July 2004-December 2008. | | | | | | | | | | | | |
| --- | --- | --- | --- | --- | --- | --- | --- | --- | --- | --- | --- | --- |
| Breast cancer flags | 3 months | | | 6 months | | | 12 months | | | 18 months | | |
|  | PPV^1^ | Sen^2^ | Spec^3^ | PPV | Sen | Spec | PPV | Sen | Spec | PPV | Sen | Spec |
| Diagnosis^4^ of invasive breast cancer | **85.9%** | **86.1%** | **99.8%** | 85.5% | 87.6% | 99.7% | **75.5%** | **88.2%** | **99.6%** | 70.5% | 88.4% | 99.5% |
| Lumpectomy | **52.0%** | **60.7%** | **99.2%** | 50.0% | 61.0% | 99.1% | **46.2%** | **61.3%** | **99.0%** | 43.5% | 61.5% | 98.8% |
| Mastectomy | **70.8%** | **32.6%** | **99.8%** | 68.8% | 34.7% | 99.8% | **62.7%** | **35.2%** | **99.7%** | 58.8% | 35.5% | 99.6% |
| Lumpectomy OR mastectomy | **56.4%** | **84.4%** | **99.1%** | 54.4% | 85.7% | 99.0% | **50.1%** | **86.3%** | **98.8%** | 47.0% | 86.4% | 98.6% |
| Lumpectomy AND diagnosis of invasive breast cancer | **89.0%** | **59.1%** | **99.9%** | 85.8% | 59.7% | 99.9% | **78.8%** | **60.0%** | **99.8%** | 73.7% | 60.2% | 99.7% |
| Mastectomy AND diagnosis of invasive breast cancer | **85.4%** | **31.8%** | **99.1%** | 81.7% | 33.6% | 99.9% | **74.7%** | **34.1%** | **99.8%** | 70.0% | 34.4% | 99.8% |
| (Lumpectomy or mastectomy) AND diagnosis of invasive breast cancer | **87.7%** | **82.3%** | **99.8%** | 82.4% | 83.7% | 99.8% | **77.2%** | **84.2%** | **99.6%** | 72.1% | 84.3% | 99.5% |
| (Lumpectomy or mastectomy) OR diagnosis of invasive breast cancer | **56.5%** | **88.2%** | **99.0%** | 54.5% | 89.7% | 98.9% | **50.1%** | **90.3%** | **98.7%** | 47.1% | 90.4% | 98.5% |
| Mastectomy OR diagnosis of invasive breast cancer | **79.7%** | **87.6%** | **99.7%** | 76.8% | 88.9% | 99.6% | **70.1%** | **89.3%** | **99.5%** | 65.6% | 89.5% | 99.3% |
| Lumpectomy OR diagnosis of invasive breast cancer | **58.2%** | **87.6%** | **99.1%** | 56.1% | 88.9% | 99.0% | **51.6%** | **89.6%** | **98.8%** | 48.5% | 89.7% | 98.6% |
| Breast radiotherapy | **55.6%** | **14.7%** | **99.8%** | 72.8% | 38.0% | 99.8% | **72.8%** | **57.6%** | **99.7%** | 66.5% | 58.1% | 99.6% |
| Dispensed medicine for breast cancer^5^ | **21.2%** | **17.8%** | **99.0%** | 36.3% | 39.3% | 99.0% | **45.5%** | **65.4%** | **98.9%** | 43.3% | 68.5% | 98.7% |

1: Positive predictive value.

2: Sensitivity.

3: Specificity.

4: Primary diagnosis field.

5: Tamoxifen, toremifene, anastrazole, exemestane, letrozole, gosrelin, trastuzumab, lapatinib, and

medroxyprogresterone 500mg.
